# Professional activity, gender and disease-related emotions: The impact on parents' experiences in caring for children with phenylketonuria

**DOI:** 10.1016/j.ymgmr.2023.100992

**Published:** 2023-07-15

**Authors:** Dariusz Walkowiak, Jan Domaradzki, Renata Mozrzymas, Dorota Korycińska-Chaaban, Monika Duś-Żuchowska, Bożena Didycz, Bożena Mikołuć, Jarosław Walkowiak

**Affiliations:** a Department of Organization and Management in Health Care, Poznan University of Medical Sciences, Poznań, Poland; b Department of Social Sciences and Humanities, Poznan University of Medical Sciences, Poznań, Poland; c Research and Development Center, Regional Specialist Hospital, Wrocław, Poland; d PKU Polyclinic, Institute of Mother and Child, Warsaw, Poland; e Department of Pediatric Gastroenterology and Metabolic Diseases, Poznan University of Medical Sciences, Poland; f Outpatient Metabolic Clinic, University Children's Hospital, Cracow, Poland; g Department of Pediatrics, Rheumatology, Immunology and Metabolic Bone Diseases, Medical University of Bialystok, Poland

**Keywords:** Rare diseases, Phenylketonuria, PKU, Caregivers, Experience, Children, Disease-related emotions, Gender, Professional activity

## Abstract

**Introduction:**

Clinical management of rare diseases often fails to acknowledge the challenges faced by caregivers. Whilst management of phenylketonuria (PKU) may not be considered as dire as other conditions, most studies primarily concentrate on clinical issues, dietary adherence, or the quality of life of the PKU patients, leaving caregivers in the background. The aim of the study was to evaluate the psychosocial effects of PKU on family caregivers.

**Methods:**

Between October 30th, 2022 and February 28th, 2023, we collected data from caregivers of children with PKU using an anonymous, self-administered, computer-assisted online questionnaire. The survey was distributed among to patients during their regular visits to five Polish PKU treatment centers.

**Results:**

A total of 159 Polish caregivers of children with PKU completed the survey. This research shows that while women caregivers were more likely to be unemployed due to their responsibilities for childcare (50.3% compared to 0% for men), and men caregivers were more likely to be employed full-time (93.8% compared to 40.6% for women), the former reported higher emotional engagement in caregiving (88.1% vs 56.3% respectively). Significantly, unemployed mothers reported higher levels of loneliness, helplessness, and emotional control problems, and lacked psychological/emotional support more often than employed mothers. This research also shows a statistically significant positive correlation between mothers' education level and financial situation (*p* < 0.05) and education level and professional activity (*p* < 0.01). Additionally, a significant positive correlation was found between perceived financial situation and feeling of happiness (*p* < 0.001), and between financial situation and professional activity (p < 0.001). Finally, a significant positive correlation was observed between feeling of happiness and professional activity (*p* < 0.05).

**Conclusions:**

According to our findings, there is a link between subjective happiness, financial situation, and professional activity among female caregivers. The relationship between these factors goes beyond just the income earned from work. The results of our study imply that there could even be a therapeutic advantage for working mothers. It is crucial to recognize the emotional difficulties that employed mothers may experience while taking care of a child with PKU, and to provide them with the necessary assistance and resources to meet their needs. Additionally, our results may provide a foundation for redefining the support system for caregivers in Poland.

## 1 Introduction

Phenylketonuria (PKU, OMIM 261600) is one of the monogenic inborn errors of metabolism, inherited in an autosomal recessive manner [1]. The lack or partial deficiency of the phenylalanine hydroxylase enzyme (PAH, EC 1.14.16.1), which transforms phenylalanine to tyrosine, is the root of the disease. When mutations occur in the PAH gene of humans, it results in PKU, with the majority of mutations linked to the misfolding and instability of the PAH enzyme [2]. An increase in the concentration of phenylalanine in the blood is a result of this deficiency, which leads to the accumulation of toxic concentrations of this substance in the brain, as well as tyrosine deficiencies. This process occurs in liver cells, using phenylalanine as a substrate, molecular oxygen, iron, phenylalanine hydroxylase, and a non-protein reaction cofactor, tetrahydrobiopterin BH4. The PAH gene, which encodes for the PAH enzyme, is located on chromosome 12 (q22-q24). Thus far, over 2200 mutations of the PAH gene, which limits the enzyme's activity in various ways, have been identified [3].

Globally, PKU affects approximately 0.45 million individuals, and its prevalence is estimated at 1 in 23,930 live births [4], although the prevalence rates differ across countries. Genetic and individual predispositions, such as the blood-brain barrier or environmental factors, contribute to the observed phenotypic heterogeneity. PKU is the most common inborn error of metabolism among Europeans. PKU was among the first disorders leading to both physical and cognitive impairments, where effective treatments were developed [5].

The primary treatment for PKU involves strict adherence to a low-phenylalanine (low-phe) diet, which involves restricting intake of foods high in phenylalanine, such as meat, dairy, and certain grains [6]. In addition to dietary restrictions, some patients may require supplementation with tyrosine, which is typically low in the PKU diet, to ensure adequate production of neurotransmitters. The strict nature of the PKU diet can make it difficult for patients and their families to maintain long-term compliance. Additionally, some individuals with PKU may still experience neurological symptoms despite strict adherence to the diet, highlighting the need for additional treatment options [7].

PKU can have a significant impact on the daily lives of individuals with the disorder and their families. The strict nature of the PKU diet can be overwhelming, especially for parents who may not have experience managing special diets. The low-phe diet requires careful attention to food choices, portion sizes, and meal planning to ensure that the child receives adequate nutrition while avoiding foods high in phenylalanine. Adherence to the required dietary restrictions can pose a challenge, restricting social activities and limiting food options [8,9]. Parents are responsible for supervising their child's diet during childhood and adolescence, which can cause them distress. This stress can be further exacerbated by various external circumstances beyond the caregiver's control [10]. Additionally, individuals with PKU may require frequent monitoring of blood phenylalanine levels and nutritional status, which can be time-consuming and costly [[11], [12], [13]]. Caregivers may also struggle with the cost of special low-phe foods and supplements, as these can be more expensive than regular foods [12,14,15].

Earlier studies have demonstrated that parents of children with chronic health condition are more susceptible to experiencing mental health disorders, psychosocial issues, and are likely to have worse health compared to parents of healthy children [16,17]. Research on the well-being of caregivers of children with PKU has produced mixed results. Some studies suggest lower level of quality of life [[18], [19], [20]], higher levels of depression and anxiety compared to the general population [16], significant psychological distress [17] while others indicate comparable or even superior well-being [21,22]. An international study found the similar quality of life scores between parents of children with PKU and the general population, but parents of children with PKU reported emotional impacts of the condition, anxiety around blood Phe concentrations, and guilt if the diet was not followed [23]. It is conceivable that the mechanisms leading to psychological distress may vary between parents of children with PKU and individuals in the general population [21]. As previously mentioned, adherence to recommendations tends to decline as the child gets older [[24], [25], [26]], even though the recommended levels of phenylalanine in the blood increase with age [6].

Economic factors also play an important role in the management and care of children with PKU [18,27,28]. There are two main reasons for this. Firstly, previous studies have highlighted that in the case of many diseases, caring for a PKU child often results in one of the parents being limited in their ability to work. Secondly, households caring for a disabled child face additional costs associated with their needs, which can be constrained by their income. These challenges include an earnings handicap, which results from barriers to employment, and a conversion handicap, which arises due to the additional and ongoing expenses associated with disability [29]. As a result, families caring for disabled children often experience a lower standard of living. Therefore, considering economic factors is important in understanding the challenges faced by families caring for a child with PKU and identifying potential interventions to improve their quality of life [11,30,31].

Understanding the factors influencing distress in parents caring for a child with PKU is crucial for providing appropriate support. Previous studies indicate that psychological resilience and social networks are important protective factors for parental well-being [32,33]. Additionally, parenting stress is predicted by child adaptive functioning, satisfaction with social support, and the challenges of meeting the child's healthcare needs [34]. Elevated parenting stress may contribute to behavioral problems in children, impacting parent-child interactions [35]. However, some research suggests that social support and child dependency may not significantly predict parental distress, while psychological resilience does. Feillet et al. discussed strategies for managing PKU beyond Phe level control, highlighting the role of psychologists in supporting families and caregivers [36]. Generally, fathers tend to have more positive well-being outcomes than mothers in parenthood [37]. However, inconsistent findings necessitate further research to better understand the impact of child dependency, social support, psychological resilience, and parental distress in PKU families [21].

Stress factors could stem from parents needing to motivate or aid their child in following their low Phe diet, as their child's growth and development rely on the diet's implementation [9]. Children become less reliant on parental management with age, and parents should support their children in transitioning to self-managing PKU [36]. However, individuals who have sustained high plasma Phe levels may experience difficulties in functioning, making this transition more challenging [38]. Moreover, we are confronted with the issue of patients who are overweight [39] [40].

Previous Polish studies on PKU primarily focused on the clinical and nutritional aspects, often overlooking the parents/caregivers. This study has two objectives. Firstly, to examine the impact of parents' professional activities on the financial situation of families with a PKU child. While dual employment generally improves a family's financial situation, we acknowledge that a parent who cares for a PKU child and does not work may receive state financial support. Thus, we aim to investigate whether non-working parents perceive their financial situation as satisfactory, considering the state support received. Secondly, we seek to determine whether employment influences the emotional well-being and subjective happiness of working parents. Is work solely a means of generating income or does it serve additional purposes? The financial status and emotional state of caregivers significantly affect the treatment outcomes of PKU children, making them vital factors in treatment success.

## 2 Methods

Data was collected from caregivers of children with PKU between October 30th, 2022 and February 28th, 2022 using an anonymous, self-administered, computer-assisted online questionnaire. The questionnaire aimed to assess the psychosocial impact of PKU on family caregivers. The study was conducted in accordance with the principles of the Declaration of Helsinki [41] and was approved by the Poznan University of Medical Sciences Bioethics Committee (KB – 833/22). Participants provided informed consent to take part in the study.

The conducted study is part of a larger project aimed at examining the problems of caregivers of children with various rare diseases. As no specific tool existed to assess the caregiving burden of caregivers of children with various RD, the survey was conducted using an original questionnaire that was developed based on a review of the literature and the study aim. The questionnaire was expanded following the guidelines of the European Statistical System [42] and was developed in consultation with a sociologist, a public health specialist, and three paediatricians specialized in metabolic disorders and management of PKU patients. The questionnaire was pilot tested using five parents, resulting in the reformulation of six questions.

It contained 26 questions designed to explore the key issues relating to problems of caregivers of children with rare diseases and were divided into several domains. The first included questions regarding concerning caregivers' demographic characteristics, including gender, education, employment, place of residence. The second domain asked questions concerning the challenges related to caring for a RD child (how they rated their child's health problems, whether they experienced problems related to the lack of psychological and emotional support, how they rated their financial situation and emotional engagement in caregiving for your PKU child, or and whether they received care allowance). The last domain focused on the emotional experiences resulting from caring for a PKU child (how often they experience such feeling as loneliness, solitude and isolation, sadness and depression, low self-esteem or helplessness, whether they feel happy and experience deterioration in your quality of life resulting from caring for a PKU child) (**Supplementary material**). The questionnaire was pilot tested using five parents, resulting in the reformulation of six questions.

The study utilized surveys administered to parents of children diagnosed with classic PKU, a condition that necessitates strict adherence to a special low-phe diet. Specialist physicians disseminated the survey link to patients during their regular visits to 5 PKU treatment centers. Moreover, the survey link was made available on websites and social media platforms through collaboration with patient and parent associations. The survey was designed to be completed within a duration of approximately 15–20 min.

The completed questionnaires were reviewed for accuracy, completeness, and consistency, and then imported into statistical software packages including JASP (version 0.17.1) and PQStat (version 1.8.4). The findings were presented using descriptive statistics. To determine differences in answer distribution among groups (gender, professional activity, place of residence), Pearson's Chi-square test with continuity correction and Fisher's exact test were used as appropriate. Kendall's rank correlation coefficient tau was utilized as a hypothesis test to assess the correlation between item scores. A 95% confidence interval (CI) for Kendall's tau was calculated using 25,000 bootstrap replicates. To assess the influence of each domicile (place of residence) stratum on the combined odds ratio (OR) of the relationship between the mother's occupational activity and the family's self-assessment of their financial situation, the Mantel-Haenszel Odds Ratio was computed, while considering the strata based on domicile. Furthermore, the homogeneity of the odds ratio across categories of the layer variable was assessed to test the hypothesis of homogeneity among all domicile strata.

## 3 Results

A total of 159 Polish caregivers of children with PKU completed the survey (Table 1). The majority of caregivers were females (89.9% vs 10.1% female caregivers), mainly mothers (mean age: 37.8 years, range: 23–72, SD = 5.6). The vast majority of respondents provided care for one PKU child (91.8%), 13 were caregiving for two or more PKU children (8.2%). Mean age of PKU children was 6.8 (range: 0.1–18, SD = 4.5). While 42.1% of caregivers reported very severe or severe health problems in their PKU children (19.5% and 22.6% respectively), the majority reported a milder form of the disease. Although most caregivers reported receiving emotional support from their families (66%), only 35.2%, reported receiving practical help in daily activities.

**Table 1:** Socio-demographic characteristics of PKU caregivers who took part in the survey.

Characteristics	N (%)
Caregiver's sex	
female	143 (89.9)
male	16 (10.1)
Caregiver's age M(SD)	
Range	23–72
M(95%CI)	37.8 (36.8–38.9)
SD(95%CI)	6.5 (5.3–7.8)
Relationship with PKU child (for PKU child I am)	
mother	142 (89.3)
father	14 (8.8)
relative (grandparent)	2 (1.3)
guardian	1 (0.6)
How many of your children experience PKU?	
1	146 (91.8)
2 or more	13 (8.2)
Child's age M(SD)	
Range	0.1–18
(95%CI)	6.8 (6.1–7.5)
SD(95%CI)	4.5 (4.1–4.9)
How would you rate you child's health problems	
very severe	31 (19.5)
severe	36 (22.6)
moderate	56 (35.2)
mild	21 (13.2)
none	15 (9.4)
While caring over my PKU child I can count on emotional support from my family	
always	66 (41.5)
often	39 (24.5)
sometimes	27 (17)
rarely	21 (13.2)
never	6 (3.8)
While caring over my PKU child I can count on practical help from my family (i.e. shopping, cleaning)	
always	28 (17.6)
often	28 (17.6)
sometimes	37 (23.3)
rarely	26 (16.3)
never	40 (25.2)
Domicile	
up to 10,000 inhabitants	70(44)
10–50,000 inhabitants	27(17)
51–100,000 inhabitants	25(15.7)
101–500,000 inhabitants	15(9.4)
above 500,000 inhabitants	22(13.9)

Women caregivers had a higher likelihood of being unemployed due to childcare responsibilities (50.3% compared to 0% for men), while men caregivers had a higher likelihood of being employed full-time (93.8% compared to 40.6% for women) (Table 2). There were no significant differences in education levels, care allowance rates, perception of financial situation, or perceived deterioration in quality of life between women and men caregivers. However, women caregivers were more likely to report a “very big” emotional engagement in caregiving (88.1% compared to 56.3% for men), while men caregivers were more likely to rate it as “big” (37.5% compared to 20.3% for women).

**Table 2:** Gender-based comparison of selected caregiving aspects for children with PKU.

	Women *N* = 143(%)	Men *N* = 16(%)	p
Professional activity			**<0.001**
unemployed	1 (0.7)	0 (0)	
unemployed due to childcare	72 (50.3)	0 (0)	
employed part-time	10 (7)	0 (0)	
employed full-time	58 (40.6)	15 (93.8)	
pension	2 (1.4)	1 (6.2)	
Education			0.81
primary or secondary	58 (40.6)	7 (43.7)	
higher	85 (59.4)	9 (56.3)	
Do you receive care allowance?			0.24
yes	101 (70.6)	9 (56.3)	
no	42 (29.4)	7 (43.7)	
How do you rate your financial situation?			0.55
very bad	8 (5.6)	0 (0)	
rather bad	27 (18.9)	1 (6.2)	
neither good nor bad	30 (21)	4 (25)	
rather good	67 (46.8)	10 (62.5)	
very good	11 (7.7)	1 (6.2)	
Do you feel happy?			0.82
never	3 (2.1)	0 (0)	
rarely	14 (9.8)	2 (12.5)	
sometimes	21 (14.7)	1 (6.2)	
often	79 (55.2)	9 (56.3)	
always	26 (18.2)	4 (25)	
Do you feel a deterioration in your quality of life?			0.44
never	20 (14)	4 (25)	
rarely	55 (38.4)	8 (50)	
sometimes	15 (10.5)	1 (6.2)	
often	41 (28.7)	3 (18.8)	
always	12 (8.4)	0 (0)	
How would you rate your emotional engagement in caregiving for your PKU child?			**<0.01**
very big	126 (88.1)	9 (56.3)	
big	14 (9.8)	6 (37.5)	
average	3 (2.1)	1 (6.2)	
little	0 (0)	0 (0)	
negligible	0 (0)	0 (0)	

Statistically significant differences are written in boldface.

**Fig. 1:**
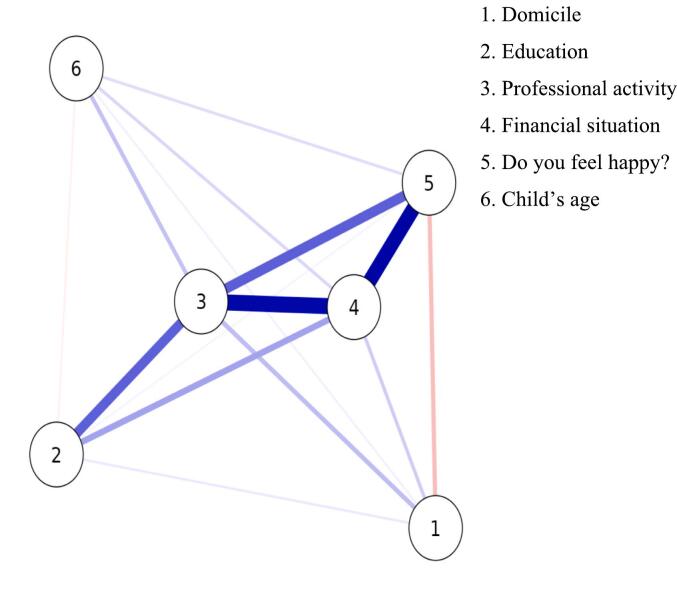
Network Analysis of Selected Sociodemographic Variables.

The results in Table 3 and Fig. 1 demonstrate the correlation coefficients (Tau B) with 95% confidence intervals (CI) and *p*-values between various sociodemographic variables and the subjective feeling of happiness. The findings reveal no significant correlations between domicile, child's age, financial situation, and professional activity with the feeling of happiness. Similarly, no significant correlations were found between caregiver's education level and the feeling of happiness, child's age, and professional activity. However, a significant positive correlation was observed between caregiver's education level and both financial situation (*p* < 0.05) and professional activity (*p* < 0.01). There were significant positive correlations between perceived financial situation and the feeling of happiness (*p* < 0.001), as well as between financial situation and professional activity (p < 0.001). Furthermore, a significant positive correlation was found between the feeling of happiness and professional activity (*p* < 0.05).

**Table 3:** Kendall's Rank Correlation Analysis of Selected Sociodemographic Variables with Subjective Feeling of Happiness.

	Tau B	95%CI	p
Domicile – Education	0.040	−0.106 -0.186	0.59
Domicile – Child's age	0.025	−0.102-0.148	0.74
Domicile – Financial situation	0.047	−0.089-0.180	0.50
Domicile – Do you feel happy?	−0.113	−0.248-0.023	0.11
Domicile – Professional activity	0.076	−0.076-0.226	0.32
Education – Child's age	0.023	−0.115-0.163	0.74
Education – Financial situation	0.148	0.001–0.298	**<0.05**
Education – Do you feel happy?	−0.005	−0.140-0.130	0.95
Education – Professional activity	0.254	0.099–0.403	**<0.01**
Child's age – Financial situation	0.026	−0.104-0.152	0.69
Child's age – Do you feel happy?	0.045	−0.094-0.184	0.49
Child's age – Professional activity	0.054	−0.088-0.195	0.45
Financial situation – Do you feel happy?	0.378	0.253–0.494	**<0.001**
Financial situation – Professional activity	0.351	0.218–0.477	**<0.001**
Do you feel happy? – Professional activity	0.200	0.050–0.343	**<0.05**

Statistically significant differences are written in boldface.

The relationships between the variables are visually depicted through the lines that connect them. Positive correlations are denoted by blue segments, while negative correlations are represented by red segments. The thickness of the line and the intensity of the color serve as visual indicators of the correlation coefficient's value, with thicker lines and more intense colors indicating stronger and more significant correlations.

Table 4 compares the emotional experiences of employed and unemployed mothers caring for a PKU child. Unemployed mothers reported higher levels of loneliness, helplessness, and emotional control problems, as well as facing problems related to the lack of psychological/emotional support, compared to employed mothers. However, there were no significant differences in low self-esteem, sadness/depression, feelings of solitude and isolation, and domicile between employed and unemployed mothers.

**Table 4:** Comparison of Emotional Experiences of Mothers Caring for a Child with PKU, Stratified by Professional Activity.

	Employed mothers *N* = 68	Unemployed mothers *N* = 73	p
Loneliness			**<0.01**
never	25 (36.8)	19 (26)	
rarely	22 (32.3)	10 (13.7)	
sometimes	11 (16.2)	24 (32.9)	
often	9 (13.2)	15 (20.5)	
always	1 (1.5)	5 (6.9)	
Low self-esteem			0.059
never	38 (55.9)	28 (38.3)	
rarely	17 (25)	14 (19.2)	
sometimes	6 (8.8)	16 (21.9)	
often	6 (8.8)	13 (17.8)	
always	1 (1.5)	2 (2.7)	
Sadness/depression			0.13
never	9 (13.2)	6 (8.2)	
rarely	23 (33.8)	22 (30.1)	
sometimes	27 (39.7)	22 (30.1)	
often	8 (11.8)	21 (28.8)	
always	1 (1.5)	2 (2.7)	
Helplessness			**<0.05**
never	4 (5.9)	4 (5.5)	
rarely	13 (19.1)	12 (16.4)	
sometimes	34 (50)	22 (30.1)	
often	15 (22.1)	25 (34.3)	
always	2 (2.9)	10 (13.7)	
Emotional control problem			**<0.05**
never	11 (16.2)	8 (11)	
rarely	14 (20.6)	14 (19.2)	
sometimes	31 (45.6)	22 (30.1)	
often	11 (16.2)	23 (31.5)	
always	1 (1.5)	6 (8.2)	
Problems related to the lack of psychological/emotional support			**<0.01**
never	18 (26.5)	6 (8.2)	
rarely	11 (16.2)	22 (30.1)	
sometimes	21 (30.9)	13 (17.8)	
often	11 (16.2)	18 (24.6)	
always	7 (10.3)	14 (19.2)	
Do you have a feeling of solitude and isolation?			0.15
never	36 (52.9)	31 (42.5)	
rarely	15 (22.1)	10 (13.7)	
sometimes	11 (16.2)	17 (23.3)	
often	4 (5.9)	12 (16.4)	
always	2 (2.9)	3 (4.1)	
Domicile			0.38
up to 10,000 inhabitants	28 (41.2)	34 (46.6)	
10–50,000 inhabitants	11 (16.2)	12 (16.4)	
51–100,000 inhabitants	11 (16.2)	12 (16.4)	
101–500,000 inhabitants	5 (7.3)	9 (12.4)	
above 500,000 inhabitants	13 (19.1)	6 (8.2)	

Statistically significant differences are written in boldface.

Given the notable variations in the labor market conditions based on the size of the locality, particularly with more favorable conditions in larger cities, the Mantel-Haenszel Odds Ratio was calculated to account for the stratification based on domicile (Table 5). The data show homogeneity (*p* = 0.09), allowing us to use the calculated odds ratio (OR = 3.44) that is consistent across all strata. This result indicates that professionally active mothers have higher odds of positive assessment of their financial situation.

**Table 5:** Odds Ratios [Mantel-Haenszel] for Positive Assessment of Financial Situation Among Mothers Stratified by Professional Activity.

Professionally active mothers vs. professionally inactive mothers	OR [MH]	95%CI	p
All domicile			
	3.44	1.72–6.91	**<0.001**
Domicile up to 10,000 inhabitants			
	6.72	2.14–21.11	**<0.001**
Domicile 10–50,000 inhabitants			
	0.86	0.16–4.47	ns
Domicile 51–100,000 inhabitants			
	1.17	0.22–6.08	ns
Domicile 101–500,000 inhabitants			
	14	0.94–207.60	ns
Domicile above 500,000 inhabitants			
	11	1.14–106.43	**<0.05**

Statistically significant differences are written in boldface.

Homogeneity of the odds ratio.

Degrees of freedom 4, Statistic = 7.95, *p* = 0.09.

## 4 Discussion

Although there have been numerous studies on the experiences of caregivers of children with PKU, it appears that this subject remains somewhat overlooked in research. To address this gap, our study represents the first comprehensive investigation in Poland focused on exploring the challenges faced by caregivers of children with PKU. Additionally, we aim to extend beyond the scope of PKU and examine the effects of caregivers' professional activity on their emotional well-being and subjective sense of happiness. We also conducted an examination of the impact of professional activity on the financial situation of the caregivers' families. By considering this aspect, we aimed to gain insights into how the employment status of caregivers influences the financial well-being of their households.

Our findings suggest that there may be potential therapeutic benefits for working mothers in the context of caring for a child with PKU. It is crucial to recognize the emotional challenges faced by employed mothers and provide appropriate support and resources to address their specific needs. It is important to note that this study did not explore the underlying reasons for the differences in emotional experiences between employed and unemployed mothers. Other factors such as work demands, financial strain, and social support networks may also contribute to these findings. These results indicate that employment status can impact the emotional experiences of mothers caring for a child with PKU. Further research is necessary to gain a better understanding of the complex relationships between employment status, caregiving responsibilities, and emotional well-being among mothers of children with PKU. Nonetheless, these findings underscore the significance of considering the unique emotional experiences of employed mothers in this context and addressing their specific needs to enhance their mental health and overall well-being.

Our results suggest that employment status may impact the emotional experiences of mothers caring for a child with PKU. Unemployed mothers may experience higher levels of loneliness, helplessness, emotional control problems, and lack of psychological/emotional support, which can be attributed to the added stress of being at home and the responsibilities of caregiving. In Poland, receiving child care allowance for PKU is associated with giving up paid work, resulting in the sacrifice of potential sources of income, including those that can be earned from home. Currently, the benefit amounts to 60% of the minimum wage in the country, and the process for qualifying and receiving this benefit is not straightforward or automatic. These regulations have been subject to debate among parents of children with various chronic health conditions, but no official decision has been made. Our study provides a valuable perspective in this discussion. Alongside the economic issues often raised by parents, our study highlights other benefits associated with the work of mothers caring for PKU children. Cultural factors influencing social roles are also noteworthy [43]. Despite the increasing emancipation of women in Poland [44], they often face a dilemma between their professional careers and caring for dependents, including children, the elderly, and the disabled. Gender relations within households are characterized by an unequal division of labor, with women assuming caregiving roles more frequently than men and having less power and authority [45]. Women with paid work are more likely than men to bear additional responsibilities such as childcare, housework, and caregiving. These expectations may force them to leave work, which can impact their life satisfaction. Work is not only a source of income but also an avenue for personal growth and can serve as a form of therapy.

Additionally, our study revealed that the perception of the financial situation is not influenced by the place of residence, which is an important finding. We obtained similar results across all five groups categorized based on the size of the locality. Working mothers, regardless of whether they reside in small towns with a lower cost of living or in large cities with more employment opportunities and higher wages, perceive the financial situation of their family significantly better than non-working mothers. This suggests that, in addition to the previously discussed benefits of their own work, the sense of contributing to the family budget may hold psychological importance.

The findings regarding the emotional states experienced by caregivers can be compared to the results of previous studies. Evans et al. did not observe significant overall differences in PKU caregivers compared to parents of healthy children, but they suggested that mothers of children with PKU might experience higher levels of emotional or subjective symptoms compared to the control group [46]. On the other hand, Gunduz et al. found that mothers of PKU patients exhibited significantly higher levels of depression and anxiety scores compared to fathers [16]. The impact of new therapies on caregivers was explored by Bosch et al., who suggested that treatments allowing for less strict adherence to the PKU diet may positively influence the quality of life for caregivers [47]. However, Feldmann argues that sapropterin treatment does not improve the health-related quality of life for parents of PKU children, as effective therapy already enables them to achieve similar results as parents of healthy children, thereby impacting their quality of life [48]. It is important to note that our research group had relatively low participation from men, indirectly supporting our findings of a lower involvement of men in the caregiving of children with PKU. Further research is needed to gain a deeper understanding of the experiences and roles of male caregivers in the context of PKU.

The gender-based comparison of selected caregiving aspects for PKU children reveals several key findings. Firstly, women were significantly more likely to be unemployed due to childcare responsibilities compared to men, while men were more likely to be employed full-time. Previous research has examined this topic and supports thenotion that the disease has a negative effect on the job prospects of at least one of the caregivers. Our study, like many others, also finds that the mother typically assumes the role of caring for the PKU child and shoulders the associated responsibilities [8,16,49,50]. Simultaneously, studies suggest that caregivers of PKU children face additional expenses related to the disease. The reduced work activity and income of one parent do not alleviate the burden of these additional costs [11,30,49,51]. Gender disparities in caregiving for children with PKU were evident in our study, with women demonstrating higher emotional engagement compared to men [52]. Mothers primarily take on the responsibility of caring for the child, including meal preparation, diet monitoring, and blood collection [53,54].

These findings highlight the need for further research and interventions that consider gender-specific factors in caregiving dynamics. Parental involvement and adaptation in managing PKU challenges are crucial for children's future development. As parents effectively manage PKU, they acknowledge it as an integral part of their lives and their child's, embracing a “new normal [55]. Understanding these unique perspectives can inform interventions and support programs to improve caregiving outcomes for families affected by PKU.

Our analysis suggests that higher education levels, better financial situations, and engagement in professional activity may be associated with an increased subjective feeling of happiness among mothers of children with PKU, as demonstrated by Iakovou and Schulpis [56]. However, their study did not find a correlation between professional activity and quality of life. Additionally, the size of the locality was found to be correlated with a negative impact on the mother's quality of life [57].

Interestingly, our findings indicate that there was no significant correlation between domicile, child's age, and the feeling of happiness. Instead, educational level, financial situation, and professional activity showed a significant positive correlation. These variables appear to be interrelated, although the underlying causes of these dependencies remain uncertain. It is worth noting that previous studies did not find a relationship between quality of life and employment status or the parent's gender. In contrast to those findings, our research highlights the importance of the child's age [58].

However, in line with prior research, a clear relationship between educational level and professional activity is evident. Nonetheless, Zengin Akkus discovered that depressive symptom scores in parents caring for children with PKU were linked to independent factors such as household income and perceived social support [59]. Similar impacts of income were reported in the study conducted by Abdelaziz [60]. Surprisingly, domicile does not seem to significantly influence professional activity and education among the respondents.

The main findings of our study emphasize the interplay between subjective happiness, financial situation, and professional activity. These results shed light on the complex relationships between sociodemographic variables and subjective well-being within the context of our study, warranting further research and analysis to better understand these correlations and their implications.

The challenges faced by parents, but also by other family members, as well as the dynamics of mutual interactions between the child and caregivers, should be recognized as equally important as the child's disease. Optimizing the functioning of the entire family should be a priority. The disease of a child can significantly impact the family's living conditions and disrupt existing patterns of functioning, potentially leading to increased tensions and conflicts among family members. It is essential to activate the adaptive capacities of the family system, if necessary with the help of specialists experienced in dealing with such challenges. A comprehensive understanding of the phenomena within the family, especially after receiving information about the child's disease, requires considering the element of time. This allows for a holistic view of the specific processes and phenomena that occur in the family, not just as cause-and-effect relationships.

To provide practical recommendations, it should be taken into consideration that:

1 Childcare care allowance should not require parents to stop working.
2 Establishment of a daycare system for PKU children should be prioritized to facilitate both parents to engage in work.
3 Establishing psychological support for mothers who are unable to work is essential and can be provided through face-to-face meetings or virtual consultations. The support should be tailored to meet the individual needs of each caregiver, and regular supervision should be in place to ensure their mental health and family's proper functioning. Incorporating these elements into the care of PKU patients can greatly benefit the caregivers and the patients they care for.

In conclusion, female caregivers were more likely to be unemployed due to childcare responsibilities, while there was no significant difference in educational attainment between male and female caregivers. Both male and female caregivers reported similar perceptions of their financial situation while caring for children with PKU. Our study found a connection between subjective happiness, financial situation, and professional activity among female caregivers. The relationship between these factors goes beyond just work-related income. The results of our study suggest that there may even be a therapeutic benefit for working mothers. It is important to acknowledge the emotional challenges that employed mothers may face while caring for a child with PKU and provide them with appropriate support and resources. Our findings may also provide a basis for redefining the support system for caregivers in Poland.

This study has several limitations that should be noted. Firstly, the survey was distributed in five Polish PKU treatment centers, resulting in a self-selected rather than random sample of respondents. Therefore, the results reflect only the opinions of those PKU caregivers who agreed to participate and cannot be generalized to the entire population of PKU parents in Poland. Secondly, there may be a potential bias associated with the online format of the survey, as not all PKU caregivers use electronic devices or may be unable or unwilling to share their experiences through this medium. Thirdly, the majority of respondents were female, mainly mothers, which introduces an implicit gender bias and may limit the perspectives presented in the results, especially those of male caregivers, including fathers, whose experiences may differ. Additionally, the lack of control data makes it challenging to draw meaningful conclusions, as the experiences of employed and unemployed mothers of non-PKU children could vary. Furthermore, the questionnaire used in the study was developed in consultation with experts but was not validated, introducing the possibility of measurement error. Consequently, caution should be exercised when interpreting the results. The study specifically focuses on the experiences of caregivers of pediatric patients with PKU and does not include the experiences of caregivers of PKU adults. This narrows the scope of the findings to a specific subgroup within the PKU population. Another limitation arises from the fact that the questionnaire was designed to assess the problems and experiences of caregivers of children with various rare diseases, potentially leading to a mismatch with the unique manifestations and challenges faced by PKU caregivers. The study also did not inquire about the timing of women caregivers' return to work, which could have influenced their experiences and reported emotions. Similarly, future studies should explore whether working mothers with unwell children under someone else's care experience heightened anxiety. Lastly, the study solely examines emotional experiences, which may discourage some parents uncomfortable with sharing personal experiences from participating in the questionnaire.

Despite these limitations, this research offers some notable advantages. Most importantly, it sheds light on the emotional experiences of PKU caregivers, a group that is often overlooked by the healthcare system in Poland. This study has the potential to inspire further research into the situation of family caregivers of PKU children. Additionally, by providing a platform for caregivers to share their experiences, it may hold therapeutic value for participants.

## Authors' contributions

DW and JD conceptualized the study. DW, JD, RM, JW and designed the research questionnaire. JD, RM, DKC, MŻ, BD, BM collected the data. DW performed the statistical analyses and prepared the tables. DW conducted the literature search and drafted the manuscript. All authors discussed the results, critically revised the article, read and approved the submitted version.

## Declaration of Generative AI and AI-assisted technologies in the writing process

Do not apply.

## Declaration of Competing Interest

The authors declare that they have no conflict of interest.

## Data Availability

Data will be made available on request.

## Appendix A Supplementary data

Supplementary material Image 1
