# Supplementary material for: Professional activity, gender and disease-related emotions: The impact on parents' experiences in caring for children with phenylketonuria
Source: Mol Genet Metab Rep. 2023 Jul 15;36:100992. doi: 10.1016/j.ymgmr.2023.100992 (PMC10368909; doi:10.1016/j.ymgmr.2023.100992)
Supplement: Supplementary file 1 — Supplementary material [file mmc1.docx]

1. Caregiver’s gender

 female

 male

2. Caregiver’s age

…….

3. Relationship with RD child

 mother

 father

 sibling

 grandparent

 legal guardian

4.What rare disease does your child experience?

…….

5. How many of your children experience RD?

 1

 2

 3 or more

6. Child’s (children’s) age

……..

7. Domicile

 up to 10,000 inhabitants

 10–50,000 inhabitants

 51–100,000 inhabitants

 101–500,000 inhabitants

 above 500,000 inhabitants

8. Education

 primary

 vocational

 secondary

 higher education

 higher medical education

9. Professional activity

 unemployed

 unemployed due to childcare

 employed part-time

 employed full-time

 pension

10. Do you receive care allowance?

 yes

 no

11. How would you rate you child’s health problems

 very severe

 severe

 moderate

 mild

 none

12. While caring over my PKU child I can count on emotional support from my family

 always

 often

 sometimes

 rarely

 never

13. While caring over my PKU child I can count on practical help from my family (i.e. shopping, cleaning)

 always

 often

 sometimes

 rarely

 never

14. How would you rate your emotional engagement in caregiving for your PKU child?

 very big

 big

 average

 little

 negligible

15. How do you rate your financial situation?

 very bad

 rather bad

 neither good nor bad

 rather good

 very good

16. Do you feel a deterioration in your quality of life?

 always

 often

 sometimes

 rarely

 never

17. Do you feel happy?

 always

 often

 sometimes

 rarely

 never

18. Do you have a feeling of solitude and isolation?

 always

 often

 sometimes

 rarely

 never

19. Do you have problems related to the lack of psychological/emotional support?

 always

 often

 sometimes

 rarely

 never

20. How often you feel lonely?

 always

 often

 sometimes

 rarely

 never

21. How often do you have low self-esteem?

 always

 often

 sometimes

 rarely

 never

22. Do you ever feel sad or depressed?

 always

 often

 sometimes

 rarely

 never

23. Do you ever feel helpless?

 always

 often

 sometimes

 rarely

 never

24. Do you ever feel problems related to the lack of psychological/emotional support?

 always

 often

 sometimes

 rarely

 never

25. Do you have emotional control problems?

 always

 often

 sometimes

 rarely

 never

26. Finally, we would like to ask: Is there anything else you would like to tell us that was not asked in this questionnaire?

……...
